# In Shift and In Variance: Assessing the Robustness of HAR Deep Learning Models Against Variability

**DOI:** 10.3390/s25020430

**Published:** 2025-01-13

**Authors:** Azhar Ali Khaked, Nobuyuki Oishi, Daniel Roggen, Paula Lago

**Affiliations:** 1 Department of Electrical and Computer Engineering, Concordia University, Montreal, QC H3G 1M8, Canada; 2 School of Engineering and Informatics, University of Sussex, Brighton BN1 9PS, UKdaniel.roggen@ieee.org (D.R.)

**Keywords:** human activity recognition, wearable sensors, deep learning, distribution shift, real world variability, data heterogeneity, model robustness evaluation

## Abstract

Deep learning (DL)-based Human Activity Recognition (HAR) using wearable inertial measurement unit (IMU) sensors can revolutionize continuous health monitoring and early disease prediction. However, most DL HAR models are untested in their robustness to real-world variability, as they are trained on limited lab-controlled data. In this study, we isolated and analyzed the effects of the subject, device, position, and orientation variabilities on DL HAR models using the HARVAR and REALDISP datasets. The Maximum Mean Discrepancy (MMD) was used to quantify shifts in the data distribution caused by these variabilities, and the relationship between the distribution shifts and model performance was drawn. Our HARVAR results show that different types of variability significantly degraded the DL model performance, with an inverse relationship between the data distribution shifts and performance. The compounding effect of multiple variabilities studied using REALDISP further underscores the challenges of generalizing DL HAR models to real-world conditions. Analyzing these impacts highlights the need for more robust models that generalize effectively to real-world settings. The MMD proved valuable for explaining the performance drops, emphasizing its utility in evaluating distribution shifts in HAR data.

## 1. Introduction

Many everyday human activities, such as walking, running, or gesturing, involve predictable physical motions. These motions can be measured by inertial measurement unit (IMU) sensors embedded in smart devices like smartphones and smartwatches. The data collected from these sensors are invaluable for Human Activity Recognition (HAR) models, which aim to classify activities automatically. HAR has a wide range of applications, including healthcare monitoring [1], sports performance analysis [2], industrial and workplace safety [3], home automation [4], and gaming [5], making it an active field of research over the past decades.

HAR models employ machine learning to classify activities. Traditional machine learning (ML) models, such as Support Vector Machines and Random Forests [6], were applied to HAR with considerable success. However, these models require manual feature engineering and domain expertise. In contrast, deep learning (DL) models, such as convolutional neural networks (CNNs) automate feature extraction [7,8,9], facilitating the classification task. Due to this ability, various DL HAR models were developed in the last decade that showed excellent results in activity classification from wearable IMU sensors on existing datasets [10,11].

However, current datasets often lack variability, as participants perform activities in controlled environments. This eliminates the real-world variability that might arise from differences between subjects, sensor positions, or devices. IMU sensors are particularly susceptible to three main sources of variability: device variability, resulting from hardware differences [12]; wearing variability, introduced by position and orientation variations when the user wears the devices [13]; and subject variability due to users performing actions in different ways [14,15].

Since variability induces a distribution shift in the data used to train and test DL HAR models, a DL HAR model must be robust to these variabilities to be reliable in healthcare, lifestyle, and pervasive health-monitoring applications. While it is known that data distribution affects the performance of DL models in general [16,17,18,19], the effects of the specific variabilities affecting IMU sensors in the performance of DL HAR models are unknown. Current DL HAR models demonstrate excellent performance on constrained and small datasets, but their robustness to various sources of data variability remains untested. Assessing DL HAR models on data with no variability provides a limited understanding of their performance and robustness. Therefore, it is crucial to understand how distribution shifts in data due to variability affect the performance of DL HAR models and how to incorporate variability into their performance evaluations.

This empirical study made the following contributions to our understanding of the effects of the variability in DL HAR models:

- We created a dataset designed specifically to study the effects of orientation, position, device, and subject variabilities in isolation from each other (Section 3.2).
- We quantified the effects of subject, orientation, position, and device variabilities using the F1 score as a performance metric (Section 3.5).
- We measured the relationship between the impact of variability and the shift in data distribution by using the Maximum Mean Discrepancy (MMD) [20] (Section 3.6).

Following this approach, we aimed to understand the data shift due to device and wearing variability and studied their effects on DL HAR models.

## 2. Related Work

Recent studies in Human Activity Recognition (HAR) demonstrated that deep learning methods, with their ability to automatically learn and extract complex features from raw data, can outperform classical machine learning algorithms [4,21]. However, distribution shifts—where the data distribution during training differs from that encountered during testing—can significantly degrade the accuracy of machine learning (ML) systems deployed in real-world scenarios [22]. These shifts can occur due to data variability caused by user behaviors, sensor placements, or changes in the device conditions over time. For instance, a HAR model trained on data from young adults might perform poorly when deployed for elderly users with different movement patterns or a HAR model trained on data from a smartwatch may perform poorly when deployed for a smart earbud. Even though these shifts are common in real-world applications, such as healthcare monitoring or fitness tracking, they are not represented in the datasets commonly used in HAR research. Current datasets often lack the variability and complexity needed to effectively evaluate the robustness of HAR models, highlighting a critical gap in existing research.

The impact of distribution shifts caused by data variability on DL models has been studied in other domains, such as image recognition and audio processing, showing significant performance degradations. Johnson and Grollmisch [23] studied the effect of the distribution shift on DL models used to classify industrial sound and found performance drops related to the data distribution changes. Distribution shifts accounted for 9–10% drops in the DL model performances. Similarly, Taori et al. [24] assessed the robustness of image classification models by evaluating models under data variability. They evaluated the robustness of the models by comparing the change in performance when testing the model with two test sets: one test had no distribution shift, and another test set had a distribution shift. Even with extensive training data, they found that the DL models showed a high susceptibility and corrupted classifications due to distribution shifts.

While these studies highlight the impact of distribution shifts on DL models in domains like image recognition and audio processing, similar challenges are also prevalent in wearable sensor-based Human Activity Recognition (HAR). The subject, wearing (orientation and position), and device variabilities can cause distribution shifts and impact the performance of models.

Subject variability is a common challenge in motion-based HAR using wearable sensors, as individuals perform activities differently [25]. Jimale and Mohd Noor [15] investigated the impact of subject variability on traditional ML HAR classification models and convolutional neural networks (CNNs). They trained their models on data collected from adults aged 19–48 and tested them on data from elderly individuals. The study found that ML models experienced an average performance drop of 9.75%, while the CNNs showed a decline of 12%, indicating that DL models may be more vulnerable to variability. Additionally, they observed changes in the features extracted for classification, suggesting that the age-related shift in the data distribution affected the model performance.

The orientation variability in HAR is highly relevant, as even for the same device and individual (smartwatches, smart earbuds), the orientation can change from day to day. Min et al. [26] found that orientation changes increased the Euclidean distance between the IMU data collected from different sessions for the same person and between different people. Yurtman and Barshan [13] found significant but varying drops in the performance of ML models when the test data was subjected to random rotation. They found that some datasets experienced a 30% drop in accuracy due to rotation, while others showed no change. The paper did not explain this varying effect of orientation variability but focused on methods to reduce its impact. Gil-Martín et al. [27] studied the effect of orientation change on CNNs. The baseline performance was obtained by training and testing the network with the original data from six public HAR datasets. The authors induced 45° of orientation changes via a matrix transformation on the test set to measure the performance after the orientation change. They found that the rotation transformation caused the model accuracy to drop by 2% to 11%, depending on which dataset was being used to train the ML models.

In a preliminary study on orientation variability and its effect on DL HAR models [28], we examined real-world orientation changes without artificial transformations. Instead, we positioned two sensors at the same location on participants, angled 45° relative to each other, which captured authentic shifts in the data distribution due to the orientation variation. The difference in model performance between these two sensor setups averaged 2%, although it varied across participants. Additionally, a negative correlation between the performance drop and the MMD changes was noted, though this trend did not hold for two of the eight participants studied.

The positional variability due to sensor placement was studied on animals in Ahn et al. [29], where changing the sensor position from the back to the neck on dogs and horses led to a significant drop in the performance of unsupervised models for Animal Activity Recognition (AAR).

Finally, Najadat et al. [30] investigated the effect of device variability on HAR classification by training a Recurrent Neural Network model using smartphones and testing it with data from smartwatches. This resulted in an accuracy of 45%, nearly half the accuracy of other scenarios based on participant-wise training–testing splits. This highlighted the impact of device variability, although it was mixed with the effect of position variability, as the smartwatches were worn on the wrist while the smartphones were worn on the waist.

In summary, subject, orientation, position, and device variabilities were identified as problems in HAR [12,15,31], but previous studies focused on only one type of variability. Even then, they were either not studied in isolation or not in the context of DL models. Moreover, the studies did not follow the same protocol to measure performance drops, which may confound multiple types of variability or other variables. This has limited our understanding of how these variabilities affect the data distribution shifts and model performance. We address this gap by analyzing the performance changes of DL-HAR models under isolated and combined variabilities and explain the performance drop using the MMD to measure the data distribution shifts.

## 3. Method

As mentioned, variability can be subdivided into subject, device, and wearing variabilities. Every variability induces a shift in the distribution of the data, leading to a change in the performance of DL HAR models. Our study aimed to characterize the effects of each type of variability in some widely used DL-HAR models. In this section, we explain the deep learning models used, the dataset collected for the evaluation, and the experimental protocol used to evaluate the effects of variability in DL-HAR models. We then explain the training settings for the models and the performance evaluation selected.

### 3.1. Deep Learning Models for HAR Evaluated

Many DL HAR models were proposed recently, and most can be categorized as homogeneous or hybrid. Homogeneous models, like those in [32,33], exclusively use deep CNN or RNN architectures. In contrast, hybrid models combine CNNs with sequential neural networks, such as RNNs [34], LSTMs [35], and GRUs [4]. Research, such as Ordóñez and Roggen [35], demonstrated that hybrid models generally exhibit superior all-around performance compared with homogeneous models. For instance, Ordóñez and Roggen [35] tested HAR classification using their proposed deep CNN with LSTM approach and found an average improvement of 6% over architectures that exclusively use deep CNNs. This improvement can be attributed to the complementary behaviors of CNNs and sequential networks. CNNs excel at extracting local features from the input and capturing spatial patterns. In contrast, sequential networks, such as RNNs, LSTMs, and GRUs, extract temporal features over the entire input window, identifying patterns and dependencies across time. Combining these strengths, hybrid models effectively leverage local and temporal information.

For this study, we chose to evaluate the robustness of three hybrid DL-HAR models: DeepConvLSTM [35], TinyHAR [36], and Attend and Discriminate [37]. These models were selected due to their unique feature extraction and temporal-information-processing approaches, as detailed in Table 1. DeepConvLSTM [35] serves as a representative model for most hybrid DL HAR models, combining CNN with LSTM to extract local and temporal features. Originally, DeepConvLSTM used two LSTM layers, as proposed by Karpathy et al. [38], but Bock et al. [21] demonstrated that a single LSTM layer performed better in most cases, which is why we used a shallow DeepConvLSTM in this study. Both Attend and Discriminate [37] and TinyHAR [36] employ attention mechanisms to improve the feature extraction, with TinyHAR additionally optimizing the model to be lightweight.

### 3.2. The Human Activity Recognition Variability (HARVAR) Dataset

For this study, we collected a dataset that highlighted the effect of variability in HAR by using multiple sensors simultaneously in varying positions and orientations and with two types of devices: Empatica Embrace Plus and Bluesense [39].

**Figure 1:**
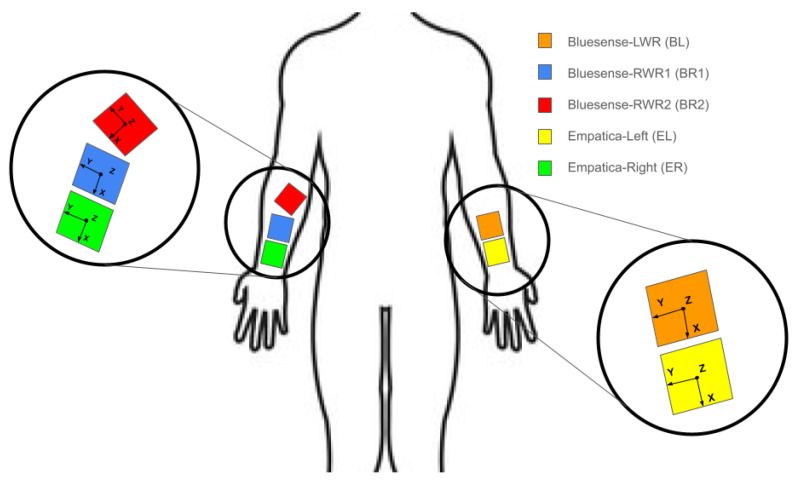
Placement of sensors in the HARVAR data collection. The Empatica Embrace Plus and Bluesense sensors were placed in the same coordinate system, and their axes are marked. BR2, marked as red, was tilted across the Z-axis at a 45-degree rotation. In this diagram, the person is facing toward the reader.

Figure 1 shows how the devices were attached to each participant. On the right wrist, participants had 3 devices: 1 Empatica (ER), 1 Bluesense (BR1) in the upright orientation, and 1 Bluesense (BR2) with a 45-degree rotation. In the left wrist, the participants had 2 devices: 1 Empatica (EL) and 1 Bluesense (BL) in the upright orientation. This information is summarized in Table 2. The sensors ER and BR1 followed the same coordinate system; similarly, BL and EL followed the same coordinate system.

Each participant performed two types of activities: treadmill walking and preparing a simple salad. The treadmill walks were conducted at speeds of 3.2 km/h, 4 km/h, 4.8 km/h, 5.6 km/h, and 6.4 km/h. Each participant walked for 2 min per speed, which meant every participant walked for approximately 10 min. On the other hand, the average time spent on the salad preparation was 20 min per participant. By collecting data simultaneously from all sensors, we eliminated the variability introduced by human factors when the same activity was performed multiple times. During the treadmill-walking phase of the experiment, the participants were not given any instructions on how to walk and were requested to walk in a way most comfortable and natural to them. Due to this, three participants chose to hold onto the support rails while walking on the treadmill, and others chose not to, as shown in Table 3.

The dataset included 16 participants from diverse age groups, with a mean age of 42 years and a standard deviation of 20 years. The data consisted of 9 male participants, with a mean weight of 74 kg and a standard deviation of 13 kg, and 7 female participants, with a mean weight of 62 kg and a standard deviation of 13 kg. The collection took place under minimal restrictions to obtain natural IMU readings. Since 30 min of labeled data were collected per participant, HARVAR had 8 h of data.

### 3.3. Experimental Protocol for Model Robustness Evaluation

**Figure 2:**
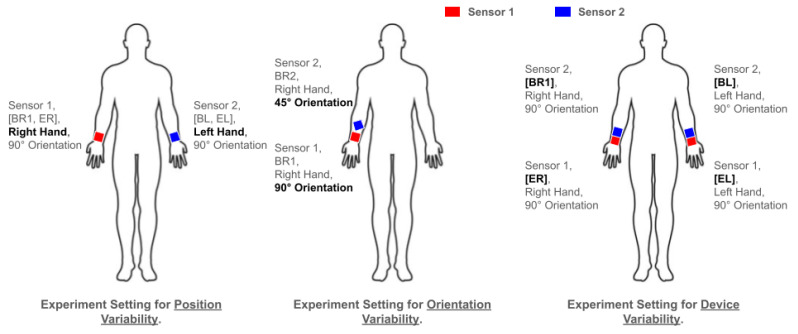
The experiment setting that used the HARVAR dataset to evaluate the effect of the device, position, and orientation variabilities, where sensor 1 and sensor 2 were used in combination as a training–testing pair to highlight the variability. In these diagrams, the person is facing toward the reader.

To understand the effect of each type of variability to be studied, we selected sensor pairs that represented the device, position, and orientation variabilities. We evaluated each model for selected pairs of sensors: once for the baseline scenario with no variability and once for the variability scenario. The pairs for the position and orientation variabilities used the same type of device on different wrists (position) or the same wrist but with rotation (orientation), respectively. In contrast, we used the same position and orientation for the device variability but different device types. These pairs are depicted in Table 4 from rows 1 to 8 and Figure 2. Notice that in each experiment, the test sensor was the same to isolate the effects of the variability, following recommendations in [22]. Testing with a different sensor would combine the effects of the different testing distributions and the variability. We evaluated the effect of variability as the performance disparity, measured with the F1 score, between the two settings.

The evaluation used cross-validation with a leave-one-subject-out (LOSO) approach in each evaluation setting. For example, for experiment 1 in the variability setting, we trained each model using the data from the Empatica-Right sensor of participants 2–16 and tested using the data from the Empatica-Left sensor of participant 1 for the first fold. This also allowed us to identify the subject variability between participants as in every baseline scenario; as shown in Table 4, we can observe the difference in performance for each participant when their data were used for testing.

### 3.4. Model Training

As mentioned, we evaluated three DL models: DeepConvLSTM, Attend and Discriminate, and TinyHAR.

We trained the models on a simple binary classification task: identifying whether or not the subject was walking. Walking was chosen because it is simple and, as shown by Xochicale et al. [14], complex motions may differ significantly between individuals. By focusing on this controlled walking activity, we aimed to minimize the impact of the motion variability, which was further mitigated through the LOSO cross-validation during testing.

The models were trained for one sensor at a time, with the input being the 3-dimensional accelerometer data. The training data were normalized in isolation from the test data using standardization and then split into sliding windows of size 2 s. The sliding windows were shuffled and split into a 9:1 ratio of training and validation. No other form of pre-processing was used on the training or validation data to maintain the originality. A weighted data loader was utilized during the training process, as the samples of the “not-walking” class outweighed the “walking” class by a 2:1 ratio. The models were trained using a batch size of 256 over 150 epochs, with early stopping called after 15 epochs of no improvement over the validation set. The initial learning rate was set to 0.001, using learning rate annealing with a patience of 7 epochs and a reduction factor of 0.1. The Adam optimizer was utilized, where it was optimized based on the CrossEntropy criterion.

Note that the model architecture remained consistent between all experiments. Still, since the Empatica and Bluesense sensors ran at different sampling rates (as shown in Table 2), the model complexity varied depending on which sensor was used to train the model. This difference in model complexity is depicted in Table 5, and the model complexity was calculated as Multiple Accumulate Operations (MACs). Bluesense sensors have a sampling frequency of 100 Hz, and Empatica sensors have a sampling frequency of 64 Hz. Since every layer in the model depends on the input size, the models trained using Bluesense sensors were more complex than those trained with Empatica.

**Figure 3:**
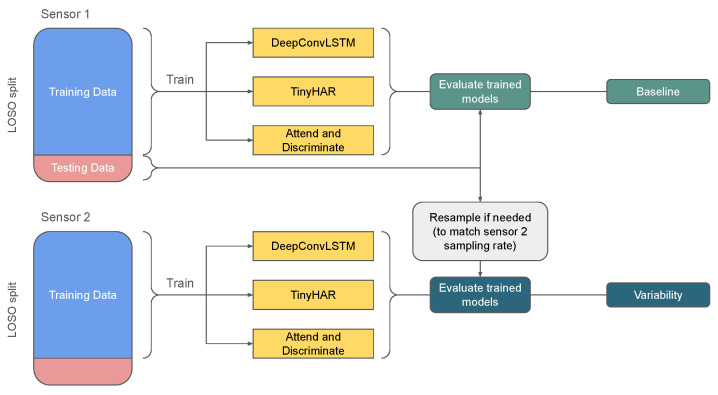
The process of evaluating the effect of variability using the HARVAR dataset.

Since we did not employ resampling before the training process, the input window size for models trained using the Empatica sensors was 128, while the input window size for models trained using the Bluesense sensors was 200. As detailed in the following subsection and shown in Figure 3, resampling was only performed using interpolation during the testing phase when the training and testing sensors had different sampling rates. Since every layer in the model depended on the input size, the models trained using Bluesense sensors were more complex than those trained with Empatica. Linear interpolation was applied to each input sliding window without frequency filtering. We saved the sliding windows for every participant and sensor to ensure consistency. This allowed us to test the model under two conditions: one with interpolation applied (device variability scenario) and another without interpolation (as the baseline scenario).

### 3.5. Model Performance Evaluation

Once the models were trained, they were tested using the data from the participants left out during the LOSO training process. Multiple tests used the data from different sensors each time, which achieved the training–testing pairs depicted in Table 4. The F1 score was used to compare the performances across various experimental settings. For the device variability, due to the different sampling frequencies of the devices, the test data were interpolated to match the sampling frequency of the training data before being input into the model. For instance, in the variability setting of experiment 5 in Table 4, the input test data were upsampled, whereas in experiment 6, the test data were downsampled. This was because in the experiment 5 variability scenario, the input data were upsampled from 64 Hz to 100 Hz, and in experiment 6, it was downsampled from 100 Hz to 64 Hz.

The significance of the difference in performance between the baseline and variability settings was tested using a *t*-test. The null hypothesis posits no difference between the baseline and variability scenarios. This hypothesis holds if the *p*-value of the *t*-test is greater than 0.05. Conversely, if there is a significant difference in performance, the *p*-value will be less than 0.05 and indicated by *, less than 0.01 by **, and less than 0.001 by ***.

### 3.6. Measuring Variability with MMD

The Maximum Mean Discrepancy (MMD) is a kernel-based statistical test used to determine the similarity between data distributions. We hypothesized that the variability introduces a distribution shift in the data, contributing to the observed effects on the performance of DL HAR models. Our study employed the multiscale kernel for the MMD with a bandwidth range of [0.2, 0.5, 0.9, 1.3, 1.5, 1.6]. A higher MMD value indicates a greater difference or shift in the data distribution, whereas a smaller MMD value suggests a smaller shift. This approach helps quantify the impact of variability on the data distribution and, consequently, on the model performance.

We calculated the MMD value between the training and testing splits used to evaluate the DL HAR models, both with and without variability. To maintain consistency in the MMD calculation, we performed the same data preprocessing steps used in the DL HAR (Section 3.4 and Section 3.5). The MMD was computed only for the labeled data, excluding null or negative classes. For the HARVAR dataset, the MMD was calculated exclusively for the walking class, omitting “not walking” activities due to their heterogeneous nature.

Typically, the MMD is calculated as a one-shot process between two data distributions, but this requires matrix multiplication and becomes computationally unfeasible for long data sequences. In our case, the walking activity spanned roughly 10 min per participant. The Bluesense sensors collected data at 100 Hz and the Empatica Embrace Plus at 64 Hz, which resulted in approximately 60,000 and 38,400 data points, respectively. Performing the MMD calculations on datasets of this size in one shot is impractical.

**Figure 4:**
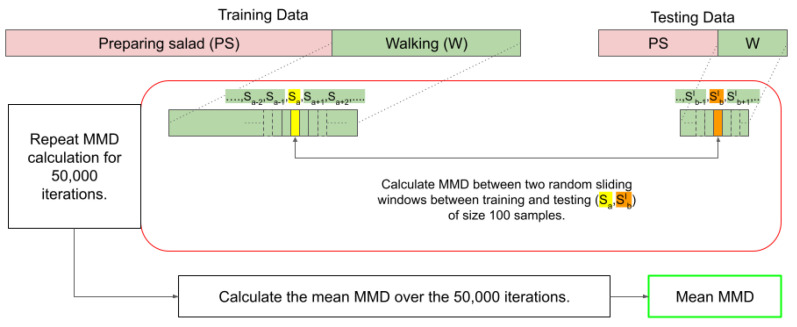
The process of calculating the MMD between the training and testing data of the HARVAR dataset.

We adopted an iterative approach to address this, as illustrated in Figure 4. We randomly selected a 100-sample window from the training and testing datasets, computed the MMD between them, and repeated this process over multiple iterations. Repeating this for 50,000 iterations, we calculated the average MMD, which we used as the final MMD value that represented the MMD between the training and testing datasets.

An iterative approach is suitable for continuous and repetitive actions like walking, as no unique artifacts in walking data are critical for classification. However, it may not be appropriate for short-duration activities, such as taking a bite, sipping water, or opening a door, where the complete time window of the activity is essential for an accurate MMD calculation. In these cases, capturing the entire activity sequence is necessary to account for specific, brief movements that are key to recognizing these actions.

### 3.7. Measuring Compounding Effects of Variability

While the HARVAR dataset allowed us to measure the effects of each type of variability in isolation, real-life scenarios often involve a combination of these variabilities. We utilized the REALDISP [40,41] dataset to study this compounding effect. The REALDISP dataset highlights the combined effect of wearing variabilities by comparing the data collected from wearable IMU sensors placed ideally by researchers (Ideal) and data from sensors worn unsupervised by participants (Self). The dataset was collected from 16 participants over two iterations for each participant. In the first attempt (Self), the participants wore the sensors without the guidance of the researchers to mimic the real-life placement of consumers who wear smart devices with IMU sensors. The second time in the “Ideal” scenario, the IMU sensors were attached to the participants by the researchers in an ideal position and orientation.

In the “Self” setting, the sensors’ position and orientation differed from the “Ideal” setting. The orientation could vary by as much as 180 degrees if worn upside down, as the sensors lacked a reference for the “correct” orientation. Unlike the HARVAR dataset, the position variability here was more subtle, as it did not involve switching the sensor from one wrist to another. Instead, the variability came from minor changes along the arm’s length. For instance, depending on the participant’s comfort, a sensor could be worn on the wrist or the forearm.

We conducted the experiments summarized in Table 6 to investigate how the wearing variability, induced by the combined effects of the position and orientation variabilities, impacted the performance of the DL models. The selected scenarios represent various training and testing conditions commonly encountered in HAR model evaluation.

The first two scenarios compared the ideal case with a variability case. In the first scenario, both the training and testing were conducted using the “Ideal” data, while in the second scenario, the “Self” data were used for training and the “Ideal” data for testing. These scenarios were analogous to experiments conducted using the HARVAR dataset, which featured a non-variability scenario (Ideal vs. Ideal) and a variability scenario (Self vs. Ideal). By comparing the performance drop between these two scenarios, we aimed to understand the effect of compounded wearing variabilities, such as orientation and position. It is important to note that this evaluation differs from HARVAR in that the HARVAR features controlled variability, where the variability was consistent across all participants. In contrast, the variability in the REALDISP dataset varied from participant to participant, as the “Self” data depended on how each participant wore the sensor.

The next two scenarios simulated real-world conditions faced when training DL HAR models. The third scenario reflected training and testing data collected in unconstrained, real-world conditions, which allowed for variability in both the training and testing. The fourth scenario represented a situation where a model was trained on lab-collected Ideal data and then deployed in real-world settings where user-induced variabilities could affect the performance (Ideal vs. Self).

Each of the four scenarios was run twice, using data from the right lower arm (RLA) and once from the left lower arm (LLA). This ensured that our results were not biased by any differences in the data caused by the dominant and non-dominant arms.

The MMD was calculated between the training and testing sets, similar to the approach used with the HARVAR dataset. However, unlike HARVAR, where we only utilized one walking activity, the REALDISP dataset included 33 different activities for classification. To calculate the MMD in this case, we computed the MMD between the training and testing sets for each activity separately, where we followed the same process as HARVAR (Figure 4). We then averaged the MMD values for each activity and quantified the distribution shift between the training and testing.

## 4. Results and Discussion

In this section, we present the results of our study. This section is organized into subsections, each exploring a research question. We begin by studying the impacts of the data variability on the model performance by isolating each type of variability in the HARVAR dataset. We then use the Maximum Mean Discrepancy (MMD) metric to explain the differences in performance across the variabilities and participants. In Section 4.3, we study the combined effects of variability using the REALDISP dataset as a more realistic scenario. We then discuss the implications (Section 4.4) and limitations (Section 4.5) of this study.

### 4.1. Variability Impacts on Model Performance

**Figure 5:**
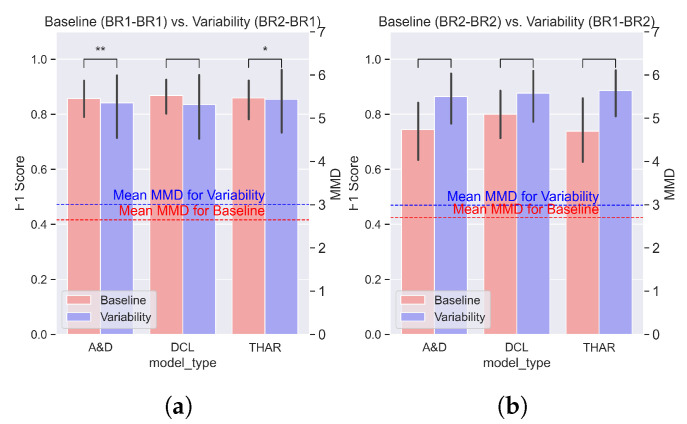
The performance changes due to the orientation variability. We show the average F1 score and average MMD values for each DL HAR model in the two experiments. The red bars represent the no variability setting of each experiment, and the blue bars represent the variability setting. The asterisks represent the *p*-value of a paired *t*-test (*: *p*-value < 0.05, **: *p*-value < 0.01, ***: *p*-value < 0.001). Only two models in one experiment showed significant performance changes, but the F1 score remained above 0.7. (**a**) The orientation variability between BR2 and BR1 when the test sensor was BR1; (**b**) the orientation variability between BR2 and BR1 when the test sensor was BR2.

**Figure 6:**
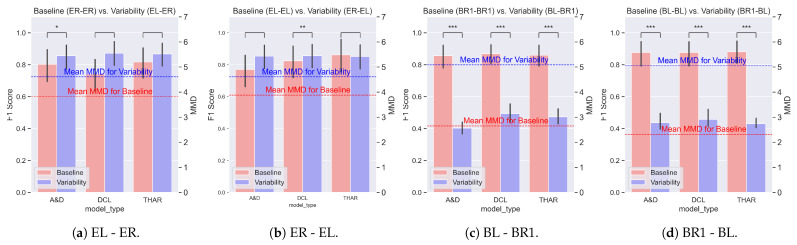
The performance changes due to the positional variability. The bars represent the average F1 score for each DL HAR model and the lines represent the average MMD values of the settings. The red bars represent the no variability setting; the blue bars represent the setting with variability. The asterisks represent the *p*-value of a paired *t*-test (*: *p*-value < 0.05, **: *p*-value < 0.01, ***: *p*-value < 0.001). Significant performance changes were found for all models when the BlueSense sensors were used but not for the Empatica sensors.

**Figure 7:**
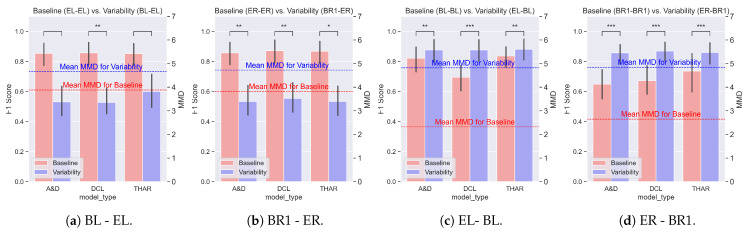
The performance changes due to the device variability. The bars represent the average F1 score for each DL HAR model, and the lines represent the setting’s average MMD values. The red bars represent the no variability setting; the blue bars represent the setting with variability. The asterisks represent the *p*-value of a paired *t*-test (*: *p*-value < 0.05, **: *p*-value < 0.01, ***: *p*-value < 0.001). Significant differences in the performance were found in all but two cases.

We evaluated the impact of the data variability on the model performance by comparing the F1 score difference on the baseline and variability settings. Figure 5, Figure 6 and Figure 7 show the averages and standard deviations of the F1 scores across all validation folds of each of the three evaluated models under no variability (blue) and orientation, position, and device variability (red) settings, respectively. The MMD values, also shown in these figures, are explained in Section 4.2.

**The orientation variability** due to the rotation of an accelerometer along one of its axes was tested using the BR1 and BR2 sensors. These sensors had a 45-degree rotation difference but were both on the right wrist. Figure 5, Figure 6 and Figure 7 show the DL HAR model’s mean F1 score for the orientation, position, and device variabilities, respectively. The mean F1 score was calculated over the F1 score acquired from all participants during the LOSO cross-validation.

Figure 5 depicts the model performance for experiments 7 and 8 in Table 4. In Figure 5a, we do not see any significant model performance changes due to the orientation variability (*p* > 0.05 in a paired *t*-test). In contrast, in Figure 5b, we see a significant drop in the performance of the Attend and Discriminate model (*p* < 0.001) and in the performance of the TinyHAR model (*p* < 0.05). We do not see a significant drop in the performance of the DeepConvLSTM model (*p* > 0.05).

In both orientation variability experiments shown in Figure 5, the performance of the baseline setting remained similar (F1 score 0.86) for all models, regardless of the test sensor. However, in the variability scenario, we saw a difference in the performance between the two experiments:

1. In Figure 5a, when the model was trained with the sensor BR2 (which was rotated 45 degrees) and tested with sensor BR1 (with no rotation), we saw no significant drop in performance. In this variability experiment, the F1 score remained above 0.81 for all three DL models.
2. In Figure 5b, when the model was trained with the sensor BR1 (which had no rotation) and tested with sensor BR2 (with 45 degrees of rotation), we saw a significant drop in the performance for the two DL HAR models (Attend and Discriminate and TinyHAR). In this variability experiment, the F1 score was less than 0.8 for DeepConvLSTM and less than 0.75 for Attend and Discriminate and TinyHAR.

**The positional variability** was evaluated across four experiments, as shown in Figure 6. The results varied depending on the sensors being used. Comparing Figure 6a,b (Empatica) with Figure 6c,d (Bluesense), we observe a greater performance drop due to positional variability when using the Bluesense sensors (mean F1 score difference of 0.45 and *p* < 0.001) than when using the Empatica sensors (mean F1 score difference of 0.12 and *p*-value close to 0.05). Since the type of variability was the same and the DL model architectures were unchanged, the larger drop in performance could be attributed to the differences between the Bluesense and Empatica sensors and how the positional variability caused a shift in their data distributions.

We note that the baseline performance of the DL models (indicated in red) was consistent and independent of the sensor used, as shown throughout the experiments in Figure 7.

The drop in performance due to the position variability was inconsistent across models when the Empatica sensors were used. In Figure 6a, we only see DeepConVLSTM show a significant drop in performance (*p* < 0.001), whereas in Figure 6b, Attend and Discriminate has the most significant drop in performance (*p* < 0.05). From the experiments performed using the Empatica sensors, we see that the DL models, in general, can be robust against positional variability for simple activities, such as walking.

**The device variability**, shown in Figure 7, caused the most significant performance drop (*p*-value < 0.001 for most cases) in the three DL HAR models compared with the position and orientation variability. The device variability experiments could be subdivided into two categories:

1. Train Bluesense and test Empatica (Figure 7a,b).
2. Train Empatica and test Bluesense (Figure 7c,d).

We see that the performance drop due to the device variability was larger for the “Train bluesense and Test Empatica” scenarios (mean F1 score drop of 0.35) vs. the “Train Empatica and Test Bluesense” scenarios (mean F1 score drop of 0.17).

**Figure 8:**
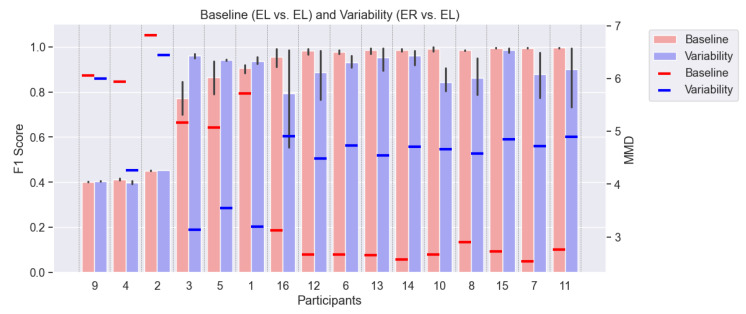
The MMD of the training vs. testing data and its relationship to the average F1 score of the three evaluated models in the ER-EL experiment. This example depicted position variability where the EL sensor data were used for testing. The blue bars represent the F1 score under the variability, the red represents the baseline F1 scores, and the blue and red points are their respective MMD values. The CVs are arranged in ascending order of the baseline F1 score.

**The subject variability** became evident when we examined the results at a granular level. Instead of just focusing on the mean performance, looking at each leave-one-subject-out cross-validation (CV) result revealed that the F1 scores for individual participants varied significantly for both the baseline and variability scenarios. Figure 8 illustrates an example of the position variability by comparing the sensors worn on the left and right wrists (Empatica-Left and Empatica-Right), detailing the F1 score per participant in the ascending order of baseline F1 scores. Out of the 16 participants, the first 6 deviated from the average trend. Participants 9, 4, and 2 exhibited very poor F1 scores of 0.4, indicating that the model’s performance was comparable with making random guesses. Participants 3, 5, and 1 performed better in the variability scenario than in the baseline scenario. All other participants followed the mean trend, where the baseline performance was higher than the performance in the variability setting.

With these results, we can observe how the **variability revealed model performance nuances**. Throughout all the experiments conducted, we observed consistent mean performances across all models in the baseline experiments. These baseline experiments mimicked the typical testing conditions for DL HAR models. Without variability, all the DL-HAR models exhibited similar high performances. However, when we isolated a specific type of variability in our experiments, we observed varying performances between the different DL models, where some models exhibited larger performance drops than others. These differences were inconsistent across the experiments, where some models had larger differences in one experiment and smaller differences in another. Evaluating the models with variability revealed their nuances and behavior under real-life conditions, demonstrating how they adapted to such changes. These results highlight the importance of testing DL HAR models under realistic conditions to better understand their robustness and adaptability.

### 4.2. Understanding Model Performance with MMD Metric

As observed in the previous section, the effect of variability in model performance was unequal across the types of variability, models, subjects, and selected test sensor. We hypothesized that the performance drop was related to the “amount of shift” in the data distribution induced by the variability. To validate this, we used the Maximum Mean Discrepancy (MMD) metric to measure the distance between two distributions.

Figure 5, Figure 6 and Figure 7 depict the average MMD for the baseline setting with a red line, and the average MMD for the variability setting with a blue line. The MMD was the same for all DL models in a given setting, as the training and testing sets were the same in each experiment. Since a higher MMD value indicates a greater shift in the data distribution, higher MMD values represent more dissimilar distributions between the testing and training data.

#### 4.2.1. MMD to Explain Orientation, Position, and Device Variabilities

We first studied the **differences in performance due to each type of variability**. Observing the average MMD values for all the experiments, it was apparent that the MMD was lower for the baseline than for the variability setting. This supports the hypothesis that the variability caused a shift in the data distribution. Moreover, the difference in the MMD between the two settings was related to the difference in the F1 score, supporting the hypothesis that the MMD was correlated with the performance.

In Figure 5, the marginal difference between the baseline and variability MMD values corresponded to the insignificant drop in the performance due to the orientation variability. Similarly, in Figure 6a,b, a small difference in the mean MMD values aligned with a small drop in the F1 score. In contrast, in Figure 6c,d, a greater difference in the MMD values corresponded to a significant drop in the DL model performance. These observations indicate a relationship between the MMD values and the performance change in the DL HAR models due to variability. The MMD difference could explain the greater performance drop when the Bluesense sensors were used as a test sensor compared with the Empatica sensors in the position variability scenarios.

Figure 7 presents a contrasting outcome to the positional variability observations in Figure 6. Here, instead of a proportional drop in the performance relative to the difference in the mean MMD values, we observed that a smaller MMD difference was related to a larger performance drop in Figure 7a,b. Conversely, in Figure 7c,d, the MMD difference was larger, but the performance drop was less significant.

This discrepancy could be attributed to the differences in the sampling rates between the sensors used in the experiments. Bluesense sensors sample at 100 Hz, while Empatica sensors sample at 64 Hz. This means that for the same 2 s time window, the Bluesense sensors provide 200 samples, whereas the Empatica sensors provide 128 samples. When a model is trained with Bluesense data (higher sampling rate) and tested with Empatica data (lower sampling rate), we must upsample the Empatica data. Upsampling does not introduce higher-frequency features into the data, which might be essential for the model’s accurate classification if trained with higher frequency information. On the other hand, if a model is trained with Empatica data and tested with Bluesense data, we downsample the Bluesense data. Downsampling removes high-frequency features from the test data, which the model, trained on lower-frequency data, does not rely on. Therefore, the performance drop is not as significant.

Another factor to consider is the model complexity. Models trained with Bluesense sensors take longer inputs for the same time window than those trained with Empatica sensors, which means that the number of inputs in each layer is larger (Table 5). More complex models may become highly specialized to the training data, which can increase their susceptibility to variability. This is because their complexity allows them to capture subtle details in the training data, which may not generalize well to data with different characteristics, leading to decreased performance when faced with variability. In contrast, less complex models might generalize better, and thus, perform more consistently under variability conditions. Further tests are required to confirm this and to explore whether more data can make the models more robust to variability. Nonetheless, it is important to remember that the amount of labeled sensor data available for HAR is usually small.

#### 4.2.2. MMD to Explain Subject Variability

We investigated the **differences in performance across participants** to highlight the subject variability. We observed a high standard deviation in the F1 score for each model, implying that each participant’s performance depended on the participant’s activity characteristics. To evaluate this, we measured the MMD for each cross-validation fold. For example, the experiment 2 position variability, as shown in Figure 8.

For participants 9, 4, and 2, who achieved F1 scores around 0.4 (indicating the model struggled to distinguish between walking and not walking), their MMD values were notably higher. This aligned with the fact that these participants held onto the support bars during the treadmill experiment, as shown in Table 3, highlighting how slight variations in activity execution could heavily impact the model performance.

Moreover, participants 3, 5, and 1 presented an exception: their baseline MMDs were higher than in the variability scenario. These participants performed better in the variability scenario but worse in the baseline scenario, which suggests that their test data in the variability setting were more similar to the training data compared with the baseline.

A consistent pattern emerged for participants 16, 12, 6, 13, 14, 10, 8, 15, 7, and 11: low MMD in the baseline setting and high MMD in the variability setting. This explains their higher performance in the baseline scenario and the drop in performance when the variability was introduced.

#### 4.2.3. MMD Correlation with F1 Score

**Figure 9:**
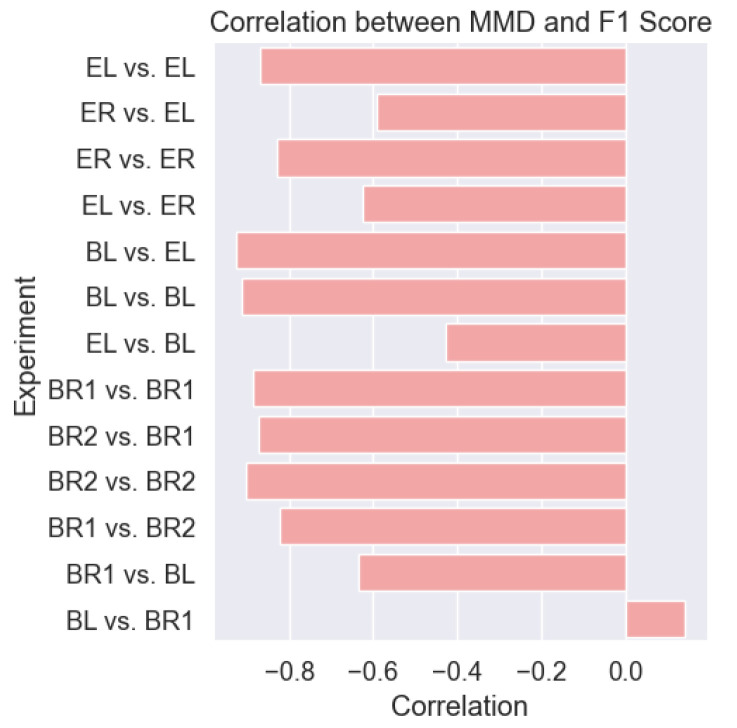
The correlation of the MMD values between the training and testing to the F1 score was mostly negative, shows there was a negative correlation between the MMD and the performance of a DL model.

Upon calculating the correlation between the F1 score and the MMD between the training and testing sets, we observed a negative correlation, as illustrated in Figure 9. This supports our hypothesis that a relationship exists between the shift in data distribution and model performance. Almost all experiments demonstrated this negative correlation between the F1 score and MMD, further validating our hypothesis.

However, an exception was found in the Bluesense-Left (BL) vs. Bluesense-Right (BR1) sensor experiment, where the correlation was closer to 0. This outlier could be attributed to the consistently poor performance of the models across all participants in the cross-validation, regardless of the MMD value. In scenarios where the model performed poorly overall, the impact of changes in the MMD appeared minimal.

We observed how introducing the variability, whether from the orientation, position, device, or subject, resulted in a higher MMD in most cases. The MMD showed how, for some participants, introducing variability helped the data become more similar to the training distribution, which explains why, in some cases, the performance increased when the variability was introduced. This relationship underscores the impact of data distribution shifts on the performance of DL HAR models and highlights the importance of considering individual participant variability in the model evaluation.

### 4.3. Compounding Variability Effects in Real-Life Scenarios (REALDISP Case Study)

**Figure 10:**
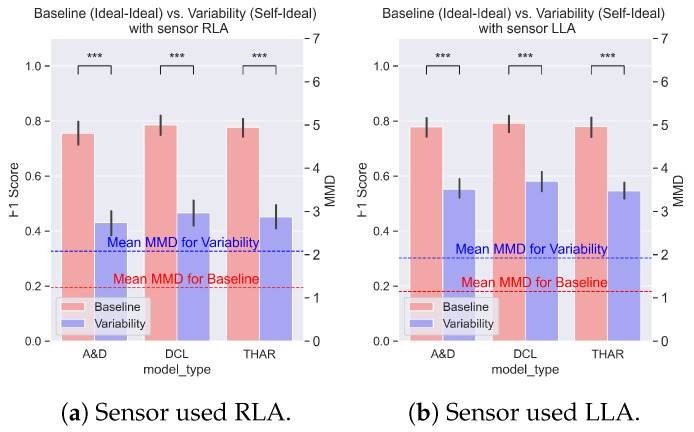
The mean F1 scores for the RLA and LLA sensors. The asterisks represent the *p*-value of a paired *t*-test (*: *p*-value < 0.05, **: *p*-value < 0.01, ***: *p*-value < 0.001). The graph shows a significant drop in performance across all three models due to variability.

The results from the REALDISP dataset revealed a significant drop in performance for both the RLA and LLA sensors due to the compounding effects of the variability (*p*-value < 0.001), as shown in Figure 10. Figure 10a illustrates the performance of the DL models trained on the data collected from the RLA sensor. Consistent with the findings from the HARVAR dataset, a higher MMD value corresponded to scenarios with a poorer performance, while a lower MMD value corresponded to scenarios with a better performance. Specifically, the MMD between the Ideal training and testing data was much lower than the MMD between the Self training and Ideal testing data.

When analyzing the performance using the LLA sensor in Figure 10b, we observed that in the Ideal vs. Ideal scenario, the DL models performed similarly regardless of whether the RLA (F1 score 0.76) or LLA (F1 score 0.78) sensor data were used. However, in the Self vs. Ideal scenario, the LLA sensor outperformed the RLA sensor. The mean F1 score for the Self vs. Ideal scenario was 0.55 when using the LLA sensor, compared with 0.44 with the RLA sensor. This difference in performance was reflected in the MMD values: the MMD for LLA-Self vs. LLA-Ideal was 1.9, while RLA-Self vs. RLA-Ideal had an MMD of 2.05. These results further confirm that a lower MMD value corresponds to better model performance, while a higher MMD value indicates a worse performance.

**Figure 11:**
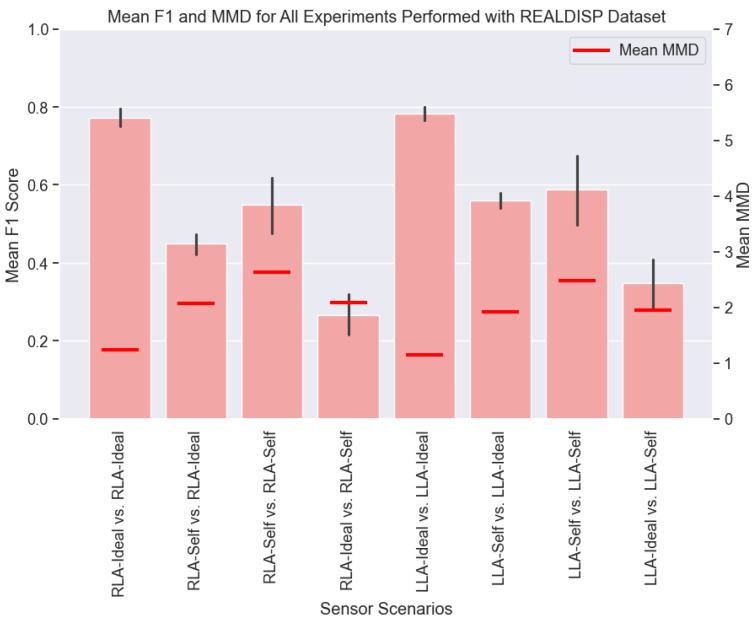
The mean F1 score and MMD values for all scenarios tested using the REALDISP dataset.

Figure 11 shows the mean F1 scores and MMD values for each scenario outlined in Table 6 for both the RLA and LLA sensors. The best performance was observed in the scenario where both the training and testing data were collected under Ideal conditions, which was expected since there was no variability to degrade the performance of the DL model. The poorest performance occurred when the model was trained on Ideal data but tested on Self collected data. This indicates that any DL HAR model trained using lab-collected data would likely perform poorly when applied in real-world settings with position and orientation variabilities.

The MMD values reveal that the highest MMD occurred when the training and testing data were of type Self. While we observed that higher MMD values generally corresponded to a lower performance, this trend did not hold in this case. The elevated MMD in the Self vs. Self scenario could be explained by the significant variability within the self data, as participants wore the sensors in varied ways, often with the sensors flipped across axes. This variability led to a wider distribution shift, which resulted in a higher MMD value. However, this diversity in the training data made the model more robust to variability, which led to better generalization and performance in the Self vs. Self scenario.

In contrast, the Ideal vs. Self scenario suffered because the models trained on Ideal data that lacked exposure to variability during the training, which made them vulnerable when tested under non-ideal conditions. Notably, despite having similar MMD values to the Ideal vs. Self scenario, the Self vs. Ideal scenario performed better. This could be attributed to the fact that when the DL HAR model was trained on diverse and variable data, it became more robust, which resulted in improved performance, even when tested on the Ideal data.

### 4.4. Implications of Results

This subsection highlights the major implications of the results of this study.

#### 4.4.1. Position and Orientation Variability Implications on Real-World Scenarios

Across the three types of variability investigated in this study, the orientation variability caused the lowest performance drops across all the models. This result suggests that models trained on IMU data from devices worn in fixed positions, such as smart glasses and earbuds, have a greater chance to generalize to multiple participants and environments, as the orientation variations that may occur will not significantly impact the performance.

Smartwatches are particularly vulnerable to the compounded effects of orientation and position variabilities. The experiments with the REALDISP dataset highlighted the significant impact of these variabilities on wrist-worn sensors, where the orientation changes could be as extreme as a 180-degree flip across an axis. This drastic orientation shift further amplified the effect on model performance when combined with the position variability. Our findings also indicate that training the models with a diverse dataset that included a range of variabilities resulted in a more robust performance, which made them better suited for real-world applications.

#### 4.4.2. Device Variability Had a High Impact on DL Model Performance

The device variability drastically impacted the performance of the DL models because it not only caused a shift in the data distribution but also introduced differences in the sampling frequency. These changes could affect the model size and necessitate resampling when using a device different from the one used in training. In addition, for this type of variability, the MMD was insufficient to understand the variability.

Given the evolving wearable device industry, device variability is one of the main challenges to truly generalizable HAR models. Currently, different models for each device are required, which means updating the models every time, which can be prohibitive if no data for the device has been collected. Researchers utilized fine-tuning and domain-adaptation methods in [42] to overcome the effect of device variability in cross-dataset scenarios, where one dataset was used to train a model and another was used to test it.

Enhancing the model robustness is crucial to addressing device variability, but it also requires careful data preprocessing and determining the optimal sampling rate for training the model. This would ensure that the model can generalize better across different devices.

#### 4.4.3. Subject Variability and the Need for Diverse Training Data

The HARVAR results show that the variability in how individuals perform activities significantly impacted the performance of the DL models. Human activity is inherently variable; these differences can change with age, demographics, and personal preferences. In our study, the participants were asked to perform a simple treadmill walking task without specific instructions, leading some to hold the side bars, while others did not. The reduced movement caused by holding the sidebars made it difficult for the models to classify walking accurately for these participants.

However, it is important to note that the training data for the DL models were not completely isolated from sidebar holding, as two participants in the training set also held onto the bars. Despite this, for CVs 9, 4, and 2, the sidebar-holding data in the training sets were outnumbered by the non-side-bar-holding data in a ratio of 2:13. This highlights the models’ bias toward the majority of the training data. To improve the performance and generalizability for larger populations, datasets must either ensure better balance across activity variations or apply preprocessing techniques that give more weight to underrepresented data in the training set.

#### 4.4.4. Larger MMD Correlated with Smaller F1 Score, with Limitations

The MMD serves as a useful metric to calculate a shift in a data distribution. We observed a strong correlation between the MMD and F1 score, namely, when the MMD was large, the F1 score was low, and vice versa. Still, it sometimes failed to fully capture the impact of the variability, as observed in the case of the device variability. When changing devices alters the input shape for a model, the MMD may not adequately explain the variability.

Additionally, the MMD is a better metric when the F1 score is high, i.e., when the model’s performance is good. However, beyond a certain threshold, when the MMD is too high, changes in the MMD stop being reflected in the changes to the performance. As seen in Figure 8, spikes in the MMD values for participants 2, 4, and 9 varied, but these three participants showed an average F1 score of 0.41. On the other hand, when the performance was high, the difference in the MMD showed a clear inverse relationship.

#### 4.4.5. No Significant Differences in Performance Change Across the Three Models

The statistical tests revealed no significant difference in the performance between the three evaluated models. However, the models with larger MACs tended to have bigger performance drops. A high model complexity results from a larger input size due to a higher sampling rate or a larger network with more layers. We assumed that increased complexity allows models to learn finer features, making them more prone to overfitting and less adaptable to changes. This aligns with previous results, such as the shallowLSTM [21] network, which showed that using one less layer in the DCL model results in a higher performance.

Models with a higher complexity require more computational resources and power. Similarly, sensors with higher sampling rates consume more power. Since the complexity of DL models for HAR increases with longer input lengths, it is more efficient to use simpler models and sensors with lower sampling rates. This approach is better suited for wearable sensors running DL HAR locally or on mobile devices, where the power and computational resources are limited. In the face of non-significant performance changes, we recommend using lighter models, such as the TinyHAR model, which achieved a similar performance and robustness with fewer parameters.

### 4.5. Study Limitations

This study isolated the effects of each type of variability in a binary classification task, while the DL models were capable of multiclass classification, as shown using the REALDISP dataset. Further studies are needed using multiclass classification with diverse activities in terms of motion and duration to better understand the robustness of the models.

We evaluated the effects of variability in two datasets, each with 16/17 participants. While these numbers are small, they are similar to other public HAR datasets. However, the small size might not be enough to reveal significant differences across models and for some experiments. Increasing the number of participants can help reveal differences across the models, but larger datasets do not showcase the same type of variabilities observed in these two datasets.

We studied wrist-worn sensor variabilities (orientation, position, and device). Future research should consider the variability in other sensor placements, such as earbuds, chest-mounted sensors, and smart glasses. The device variability was only tested between two devices with 64 Hz and 100 Hz sampling frequencies, while many other devices with different noise levels and sensitivity ranges exist. As this type of variability showed the highest drops in performance, a deeper study on its effects and how to overcome it might be required. Other research [43] also found that the sampling rate of the training and testing data should match for an optimal performance.

Finally, we focused solely on accelerometer data, whereas many DL models are designed to combine multiple modalities, including gyroscope and magnetometer data, for HAR. We used only the accelerometer, as it is the most common modality in many devices and has the lowest power consumption, making it preferable when possible.

## 5. Conclusions

In this study, we investigated four types of variability in three different DL-HAR models. We isolated each type of variability in our experiments, which was performed with the HARVAR dataset, a dataset specifically collected for this study. We evaluated the distribution and performance changes caused by the position, orientation, and device changes.

Our findings highlight that different types of variability affect DL HAR models in distinct ways. The orientation variability had the least impact on the DL HAR models, whereas the position and device variability resulted in significant performance drops. The subject variability was seen within the performance discrepancy from one participant to the other based on how they performed the activity. The impact of variability on the DL HAR models also depended on the sensors used. For instance, the position variability had a greater effect when using the Bluesense sensors than the Empatica sensors.

Our study showed a direct relationship between the changes in MMD values and the drop in the DL model performance, emphasizing that a higher shift in the data distribution (as indicated by the MMD) corresponded to a lower performance (F1 score). We recommend using the MMD to predict potential performance drops when switching a model trained on a specific position, orientation, or device to another. Although the MMD may not provide a complete picture, it is a useful metric for estimating performance changes.

We found that the subject variability significantly impacted the performance of all three DL models. Variations in how activities are performed, especially for more complex activities than walking, can greatly affect the model accuracy. This raises concerns about the reliability of DL models when tested on small, constrained datasets collected in controlled lab environments, where participants follow meticulously prescribed routines. Such settings may not capture the variability seen in real-world scenarios, questioning the generalizability of these models.

Using a sensor with a higher sampling rate increased the input size for the DL HAR models, which increased their complexity. The models with higher MACs tended to perform worse in the presence of variability than the models with lower MACs, as seen in the device variability scenarios. This suggests that a high sampling rate may lead the model to rely on nuanced high-frequency features, which diminishes the model’s ability to handle variability effectively. Additionally, when comparing the models, namely, DeepConvLSTM, TinyHAR, and Attend and Discriminate, we found that a higher model complexity, as seen in DeepConvLSTM and Attend and Discriminate, did not necessarily translate to better performance in handling the variability. Therefore, we recommend using lighter models like TinyHAR, which consistently performed well despite the variability.

Device variability was studied before in traditional machine learning models, but its impact on DL models is unique. Since the complexity of DL models depends on the size of the input window, our study found that higher sampling rates are often unnecessary, as human activities generally do not involve high-frequency movements. Additionally, higher sampling rates can lead models to focus on finer details of the input data, causing overfitting and poor generalizability. This is not an issue in traditional machine learning models, as they rely on features rather than raw IMU sensor data for HAR.

This study aimed to highlight the impacts of various real-world variabilities on DL HAR models and examine their isolated effects. We analyzed the influence of three isolated variabilities on a simple activity, like walking, using the HARVAR dataset. Then, we demonstrated the effect of compounded variability across 33 different activities using the REALDISP dataset. A limitation of our work was that we did not isolate the effect of variability on activities other than walking, and our data was limited to 16 participants. Our findings suggest that incorporating a diverse range of variability in the training data enhanced the robustness of the DL models, as evidenced by the results from REALDISP. Utilizing the MMD as a metric for data distribution shifts and the training–testing pipeline developed in this research can enable future studies to evaluate the robustness of DL HAR models beyond ideally collected datasets.

## Figures and Tables

**Table 1:** The three SOTA DL HAR models used for this study and their key architectural differences.

DeepConvLSTM	Attend and Discriminate	TinyHAR
Feature extraction (extraction of local or short-time features):		
Four 1-dimensional convolution layers with a kernel size of 5, a stride of 2, and 64 filters were used to extract local features from the input data.	Local feature extraction was achieved in two steps: First, the data were processed through 4 one-dimensional convolution layers with a kernel size of 5, stride of 2, and 64 filters. Second, a transformer encoder block that comprised a self-attention and two fully connected feed-forward layers encoded the channel interaction, where the channels were the readings from various sensor modalities.	The local feature extraction was achieved in three steps: First, the data were processed through 4 one-dimensional convolution layers with a kernel size of 5, stride of 2, and 20 filters. Second, a transformer encoder block that comprised a self-attention and two fully connected feed-forward layers encoded the channel interaction. Third, a fully connected layer fused the cross-channel interaction information.
Temporal information extraction (extraction of features over the entire time window):		
A single LSTM layer with 128 cells was used to extract the temporal features.	The temporal information was extracted in two steps: First, a single GRU layer with 128 cells extracted the temporal features. Second, a self-attention layer was used to highlight the important temporal features.	The temporal information was extracted in two steps: First, a single LSTM layer with 40 cells extracted the temporal features. Second, a self-attention layer was used to highlight the important temporal features.

**Table 2:** The sensors used for the collection of the HARVAR dataset, along with information on their sampling rates and placements.

Sensor Type	Sensor Position	Sensor Name	Sensor Code	Sampling Frequency
Bluesense	Right wrist (no rotation)	Bluesense-RWR1	BR1	100 Hz
Bluesense	Right wrist (45° rotation)	Bluesense-RWR2	BR2	100 Hz
Bluesense	Left wrist	Bluesense-LWR	BL	100 Hz
Empatica	Right wrist	Empatica-Right	ER	64 Hz
Empatica	Left wrist	Empatica-Left	EL	64 Hz

**Table 3:** Information about the 16 participants of HARVAR dataset with notes on how they performed the treadmill walk.

ID	Age	Sex	Weight (kg)	Holding Sidebar
1	59	M	83.9	No
2	74	F	65.7	Yes
3	60	F	49.8	No
4	71	M	79.0	Yes
5	61	F	55.3	No
6	71	M	64.8	No
7	26	F	73.0	No
8	25	M	72.5	No
9	26	M	61.0	Yes
10	47	M	89.8	No
11	23	F	53.0	No
12	21	M	55.0	No
13	24	F	74.8	No
14	35	F	86.2	No
15	29	M	73.0	No
16	26	M	95.0	No

**Table 4:** List of experiments conducted using the HARVAR dataset.

Exp. ID	Variability Type	Training Sensor	Testing Sensor	Setting
1.	Position	Empatica-Right	Empatica-Left	Variability
		Empatica-Left	Empatica-Left	Baseline
2.	Position	Empatica-Left	Empatica-Right	Variability
		Empatica-Right	Empatica-Right	Baseline
3.	Position	BRW1	BLW	Variability
		BLW	BLW	Baseline
4.	Position	BLW	BRW1	Variability
		BRW1	BRW1	Baseline
5.	Device	BLW	Empatica-Left	Variability
		Empatica-Left	Empatica-Left	Baseline
6.	Device	Empatica-Reft	BLW	Variability
		BLW	BLW	Baseline
7.	Orientation	BRW1	BRW2	Variability
		BRW2	BRW2	Baseline
8.	Orientation	BRW2	BRW1	Variability
		BRW1	BRW1	Baseline

**Table 5:** The computational complexity (in MACs) variance between the different models. The computational complexity depended on the model architecture and the sensor being used to train due to the difference in the sensor sampling rates.

Model	Sensor	Computational Complexity (MACs)	Parameters
TinyHAR	Bluesense	$2.53\times 10^{6}$	24,864
DeepConvLSTM	Bluesense	$2.19\times 10^{7}$	227,138
Attend and Discriminate	Bluesense	$8.36\times 10^{7}$	297,412
TinyHAR	EmpaticaEmbrace+	$1.53\times 10^{6}$	24,864
DeepConvLSTM	EmpaticaEmbrace+	$1.33\times 10^{7}$	227,138
Attend and Discriminate	EmpaticaEmbrace+	$5.12\times 10^{7}$	297,412

**Table 6:** Experiments to evaluate the compound effects of variability using the REALDISP dataset.

Exp. ID	Scenario	Train Data	Test Data	Sensor
1.	Lab scenario, trained and tested with Ideal data	Ideal	Ideal	RLA/LLA
2.	Trained with variable data and tested with Ideal data	Self	Ideal	RLA/LLA
3.	Trained and tested with variable data	Self	Self	RLA/LLA
4.	Lab trained and tested on variable data	Ideal	Self	RLA/LLA

## Data Availability

The datasets presented in this article are not readily available because it is a part of an ongoing study. Requests to access the datasets should be directed to azharali.khaked@mail.concordia.ca or paula.lago@concordia.ca.
